# A cohort study of 19 patients with gyrate atrophy of the choroid and retina (GACR)

**DOI:** 10.1007/s00417-024-06540-8

**Published:** 2024-06-07

**Authors:** Berith M. Balfoort, Filip Van Den Broeck, Marion M. Brands, Clara D. van Karnebeek, Arthur A. Bergen, L. Ingeborgh van den Born, Riekelt H. Houtkooper, Margreet A. E. M. Wagenmakers, Julie De Zaeytijd, Bart P. Leroy, Camiel J. F. Boon, Roselie M. H. Diederen

**Affiliations:** 1 Department of Paediatrics, Emma Children’s Hospital, Amsterdam UMC, University of Amsterdam, Amsterdam, Netherlands; 2 Laboratory Genetic Metabolic Diseases, Amsterdam UMC, University of Amsterdam, Amsterdam, Netherlands; 3 Department of Ophthalmology, Ghent University Hospital, Ghent, Belgium; 4 Department of Ophthalmology, Amsterdam UMC, University of Amsterdam, Meibergdreef 9, 1105 AZ Amsterdam, Netherlands; 5 Department of Human Genetics, Section Ophthalmogenetics, Amsterdam UMC, University of Amsterdam, Amsterdam, Netherlands; 6 Emma Center for Personalized Medicine, Amsterdam UMC, Amsterdam, Netherlands; 7 Department of Internal Medicine, Centre for Lysosomal and Metabolic Diseases, Erasmus MC, Erasmus University Medical Centre Rotterdam, Rotterdam, Netherlands; 8 The Rotterdam Eye Hospital, Rotterdam, Netherlands; 9 Department of Ophthalmology, Leiden University Medical Centre, Leiden, Netherlands; 10 Amsterdam Gastroenterology, Endocrinology and Metabolism, Amsterdam UMC, University of Amsterdam, Amsterdam, Netherlands; 11 Department of Head & Skin, Ghent University, Ghent, Belgium; 12 Centre for Medical Genetics, Ghent University Hospital, Ghent, Belgium

**Keywords:** OAT, Retinal degeneration, Inherited retinal disease, Ornithine, Gyrate atrophy

## Abstract

**Purpose:**

Gyrate atrophy of the choroid and retina (GACR) is an autosomal recessive inherited metabolic disorder (IMD) characterised by progressive retinal degeneration, leading to severe visual impairment. The rapid developments in ophthalmic genetic therapies warrant knowledge on clinical phenotype of eligible diseases such as GACR to define future therapeutic parameters in clinical trials.

**Methods:**

Retrospective chart analysis was performed in nineteen patients. Data were analysed using IBM SPSS Statistics version 28.0.1.1.

**Results:**

Nineteen patients were included with a mean age of 32.6 years (range 8–58). Mean age at onset of ophthalmic symptoms was 7.9 years (range 3–16). Median logMAR of visual acuity at inclusion was 0.26 (range -0.18–3.00). Mean age at cataract surgery was 28.8 years (*n* = 11 patients). Mean spherical equivalent of the refractive error was -8.96 (range -20.87 to -2.25). Cystoid maculopathy was present in 68% of patients, with a loss of integrity of the foveal ellipsoid zone (EZ) in 24/38 eyes. Of the 14 patients treated with dietary protein restriction, the four patients who started the diet before age 10 showed most benefit.

**Conclusion:**

This study demonstrates the severe ophthalmic disease course associated with GACR, as well as possible benefit of early dietary treatment. In addition to visual loss, patients experience severe myopia, early-onset cataract, and CME. There is a loss of foveal EZ integrity at a young age, emphasising the need for early diagnosis enabling current and future therapeutic interventions.

**Supplementary Information:**

The online version contains supplementary material available at 10.1007/s00417-024-06540-8.


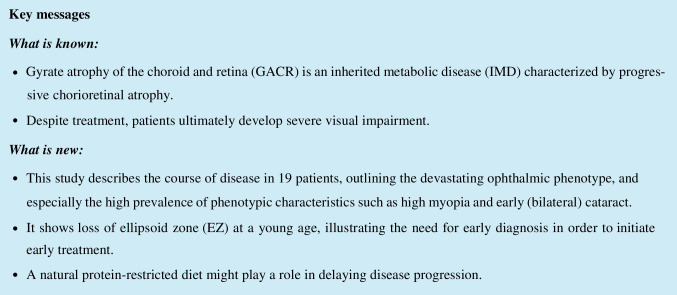


## Introduction

Gyrate atrophy of the choroid and retina (GACR) (OMIM #258,870) is an inherited metabolic disease (IMD) with primarily an ophthalmic phenotype. The predicted global prevalence of GACR is 1:1,500,000. Due to a founder effect, prevalence in Finland is 1:50,000 [1].

Patients typically present in the first decade of life with symptoms as myopia and nyctalopia [2]. As the disease progresses, patients experience concentric visual field loss due to progressive chorioretinal atrophy. Disease progression can be visualised on fundoscopy as round and sharply demarcated atrophic lesions, initially outside the macula. Over time, these lesions coalesce into large atrophic zones [3]. A rare first clinical presentation is that of neonatal hyperammonaemia due to reversed ornithine flux in the first few weeks of life. In these cases, diagnosis is usually made before the development of ophthalmic symptoms [4].

It has been previously thought that central vision remains intact for several decades, although it can be severely affected by complications such as early (bilateral) cataract, cystoid maculopathy, and macular neovascularisation [3]. There is a large degree in phenotypic heterogeneity among patients with GACR although this might be dependent on therapy and therapy compliance [5].

GACR is caused by pathogenic biallelic variants in *OAT*, encoding mitochondrial matrix enzyme ornithine-δ-aminotransferase (OAT). OAT catalyses the reversible reaction of ornithine and α-ketoglutarate to glutamate and glutamate-5-semialdehyde (GSA) but is mainly involved in ornithine catabolism after the neonatal phase [6]. Therefore, OAT deficiency leads to the accumulation of ornithine in all bodily fluids. It is unclear why the eye is specifically affected.

Currently, there are no curative therapies available for GACR. Most therapeutic interventions aim to lower plasma ornithine, primarily with a natural protein-restricted diet, where natural refers to proteins present in food. With a natural protein-restricted diet, ornithine precursor arginine is restricted in intake, leading to a decrease in plasma ornithine. This therapy adheres to the hypothesis that hyperornithinaemia might be toxic to the delicate structures of the eye [7, 8]. To prevent deficits, the natural protein-restricted diet is enriched with supplements containing essential amino acids and no arginine.

With the rapid developments of genetic therapies for inherited retinal diseases [9, 10], it is logical to consider GACR as a potential candidate disease for such an intervention. Due to its monogenetic nature and distinct ophthalmic phenotype, GACR is a suitable target for genetic ophthalmic interventions, as systemically correcting plasma ornithine might not be sufficient as a sole therapeutic intervention [11].

However, inclusion criteria and optimal time for genetic intervention for GACR need to be defined, which requires an optimal understanding of the disease, including insight in genotype–phenotype correlations, phenotypic variability, and disease progression [12]. As the phenotype has mainly been described in case report or small case series, and ophthalmic assessment and follow-up is highly variable and does not always compare well, there is a need for larger cohort studies.

This study describes a cohort of 19 GACR patients and delineates the ophthalmic GACR phenotype and associated complications. Our objective is to provide the clinician with a better understanding of the disease and management. Ultimately we hope to enhance trial readiness, serving as a stepping stone for more extensive retrospective and prospective natural history studies in anticipation of future therapeutic trials.

## Methods

### Data collection

The study protocol of the Gyrate Atrophy Registry was approved by the Medical Ethical Committee of the Amsterdam University Medical Centres (No. 21.310), the Netherlands, and the Ethical Committee of Ghent University Hospital, Belgium. Patients were included after written informed consent was obtained from patients and/or legal guardians. The study adhered to the tenets of the Declaration of Helsinki.

Patients were included if previously diagnosed with GACR, which was defined as the combination of the typical retinal lesions described by an ophthalmologist and hyperornithinaemia and/or biallelic pathogenic variants in *OAT*.

### Clinical examination

For all patients, retrospective data were extracted from patient files. Extracted data included BCVA, visual fields (HFA), refractive errors, and retinal imaging, including fundus photography and SD-OCT. Last measurement of refractive errors before cataract surgery was used for analysis in those patients that were pseudophakic. Age of onset of ophthalmic symptoms was defined as the age at which the patient first reported ophthalmic symptoms, including those of myopia.

Patients residing in the Netherlands were invited to visit the Amsterdam UMC expertise centre outpatient clinic to be assessed according to a standardised ophthalmic protocol. Best corrected visual acuity (BCVA) was measured using the Snellen visual chart. Visual fields were examined using automatic static perimetry with the Humphrey Field Analyser (HFA; Carl Zeiss Meditec, Inc. Dublin, CA, USA). Foveal sensitivity and mean deviation (MD) in dB were used as outcome measures of HFA visual field analysis [13], in addition to an estimation of residual visual field in degrees.

Retinal imaging included fundus photography (Topcon TRC-50DX, Topcon Medical Systems Inc), spectral-domain optical coherence tomography (SD-OCT; Spectralis), and short-wavelength (488 nm) fundus autofluorescence (FAF; Heidelberg Engineering).

SD-OCT images of all patients were assessed for the presence of cystoid maculopathy and/or the presence of an epiretinal membrane. Cystoid maculopathy was defined as fovea-involving or non-fovea-involving. In addition, ellipsoid zone (EZ) integrity was assessed visually on SD-OCT images. A distinction was made between perifoveal and foveal EZ. EZ was defined as continuous, discontinuous, or indiscernible [14]. SD-OCT images were assessed by two independent graders (B.M.B. and R.M.H.D.) and reviewed by a third grader (C.J.F.B.) in case of discrepancy.

### Statistical analysis

Data analysis was performed using IBM SPSS Statistics version 28.0.1.1 (IBM Corp. Armonk, NY, USA). Continuous data are presented as mean ± SD or range, in case of normal distribution, and as median, interquartile range (IQR), and range, in the case of non-normal distribution. Categorical data are presented as frequencies and percentages. Normality was tested using the Shapiro–Wilk test and by plotting the data.

Decimal VA scores were converted to logMAR using the formula logMAR = -log(decimal acuity). The spherical equivalent (SE) of the refractive error was calculated using the standard formula: SE = sphere + ½ cylinder. Correlation testing was performed with the Spearman or Pearson correlation test, depending on normality of data. A *p*-value < 0.05 was deemed statistically significant.

## Results

### Clinical, biochemical, and genetic characteristics

In total, 19 patients from 16 different families were included in this study (38 eyes) (Table 1). Sixty-three percent of patients (*n* = 12) were male. The mean age at inclusion was 32.6 years (standard deviation [SD]: 15.9, range 8–58). Eighteen patients had a molecular diagnosis confirming the diagnosis of GACR; one patient was diagnosed based on hyperornithinaemia and a molecular diagnosis in an older sibling. Eleven different pathogenic variants in *OAT* were found, the majority being missense variants (*n* = 9). Other variants were frameshifts caused by either a nucleotide duplication (*n* = 2) or nucleotide deletion (*n* = 1).

**Table 1:** Patient characteristics of 19 patients included in the Gyrate Atrophy Registry

ID	Sex	Ethnicity	Age at inclusion	Age of onset ophthalmic symptoms	Age at diagnosis	Symptoms at diagnosis	*OAT p*athogenic variant #1	*OAT p*athogenic variant #2	Type of genetic testing	Plasma ornithine at diagnosis (µmol/L)
P1	Male	Sri Lankan	37	3	15	Myopia	c.1A > G; p.(1 M)	c.1A > G; p.(1 M)	OAT sequencing	636
P2	Female	Dutch	45	6	12	Myopia	c.1058G > A; p.(Gly353Asp)	c.1058G > A; p.(Gly353Asp)	OAT sequencing	988
P3	Female	Turkish	25	9	2	Neonatal hyperammonaemia	c.1276C > T; p.(Arg426Ter)	c.1276C > T; p.(Arg426Ter)	OAT sequencing	399
P4	Male	Dutch	13	3	5	Myopia, nyctalopia	c.1058G > A; p.(Gly353Asp)	c.460C > T; p.(Arg154Cys)	OAT sequencing	876
P5	Female	Finnish	17	7	14	Myopia	c.539G > C; p.(Arg180Thr)	c.539G > C; p.(Arg180Thr)	OAT sequencing	819
P6	Female	Finnish	19	9	17	PFH, myopia	c.539G > C; p.(Arg180Thr)	c.539G > C; p.(Arg180Thr)	OAT sequencing	770
P7	Female	Dutch	57	7	10	Myopia	c.1058G > A; p.(Gly353Asp)	c.1034 T > C; p.(Leu345Pro)	OAT sequencing	935
P8	Male	Dutch	31	16	16	PFH, myopia	c.1058G > A; p.(Gly353Asp)	c.1112dup; p.(Arg372Lysfs*12)	OAT sequencing	859
P9	Male	Dutch	24	8	8	Myopia, nyctalopia	c.1058G > A; p.(Gly353Asp)	c.1112dup; p.(Arg372Lysfs*12)	WES	721
P10	Female	Dutch	21	5	6	Myopia	c.1058G > A; p.(Gly353Asp)	c.1172delT; p.(Trp391Glyfs*29)	NGS panel	805
P11	Male	Dutch	40	6	25	Nyctalopia, progressive vision loss, PFH, myopia	c.1058G > A; p.(Gly353Asp)	c.460C > T; p.(Arg154Cys)	OAT sequencing	737
P12	Female	Dutch	33	7	32	Nyctalopia, myopia, dry eyes	c.1058G > A; p.(Gly353Asp)	c.1058G > A; p.(Gly353Asp)	WES	939
P13	Male	Turkish	12	12	0	Neonatal hyperammonaemia, PFH	*c.1276C* > *T; p.(Arg426Ter)*^+^	*c.1276C* > *T; p.(Arg426Ter)*^+^	NA	56
P14	Male	Dutch	49	10	40	Nyctalopia, myopia, cataract, progressive vision loss	c.1058G > A; p.(Gly353Asp)	c.1058G > A; p.(Gly353Asp)	NGS panel	805
P15	Male	Indo-Surinamese	28	14	28	Myopia	c.1192C > T; p.(Arg398*)	c.1235 T > C; p.(Ile412Thr)	NGS panel	653
P16	Male	Belgian	8	4	7	Myopia	c.1058G > A; p.(Gly353Asp)	c.1058G > A; p.(Gly353Asp)	WES	694
P17	Male	Italian, Romanian	58	Unknown	23	Unknown	c.1173G > A; p.(Trp391Ter)	c.1173G > A; p.(Trp391Ter)	WES	1086
P18	Male	Italian, Romanian	52	12	12	PFH, myopia	c.1173G > A; p.(Trp391Ter)	c.1173G > A; p.(Trp391Ter)	WES	617
P19	Male	Belgian	50	12	Unknown	Vision loss	c.1058G > A; p.(Gly353Asp)	c.1058G > A; p.(Gly353Asp)	OAT sequencing	917

*PFH = positive family history*. WES* = Whole Exome Sequencing*. NGS* = Next Generation Sequencing*. NA* = not applicable*.* + *No pathogenic variants were available for this patient, but it is likely to have the same genotype as full sibling P3*

The mean age at onset of ophthalmic symptoms was 7.9 years (SD 3.8, range 3–16. The mean age at diagnosis was 15.2 years (SD 10.7, range 1–40). The mean number of years until a diagnosis was established was 6.8 (SD 10.7, range -11–30). Negative values are of patients that were diagnosed prior to the development of ophthalmic symptoms. Common first ophthalmic symptoms were myopia (*n* = 15) and nyctalopia (*n* = 5). Five patients were diagnosed through family screening after a diagnosis in a sibling.

At inclusion, mean plasma ornithine was 686.7 µmol/L (SD 251.0, range 261–1013), compared to 792.0 µmol/L (SD 161.24, range 399–1086) at diagnosis (normal values: 27–98 μmol/L). P13 was excluded from analysis of diagnostic values as this patient was diagnosed during an episode of neonatal hyperammonaemia with a normal plasma ornithine. After the neonatal phase, plasma ornithine rapidly increased.

### Visual acuity, refraction, cataract, and visual field

The median logMAR of visual acuity at inclusion was 0.26 (IQR 0.73, range -0.18–3.00). This corresponds to a mean decimal acuity of 0.57 (SD 0.39, range 0.001–1.50). Spearman’s ρ defined a significant correlation between the age at inclusion and corresponding logMAR values (*p* < 0.001) with a correlation coefficient of 0.685.

**Fig. 1:**
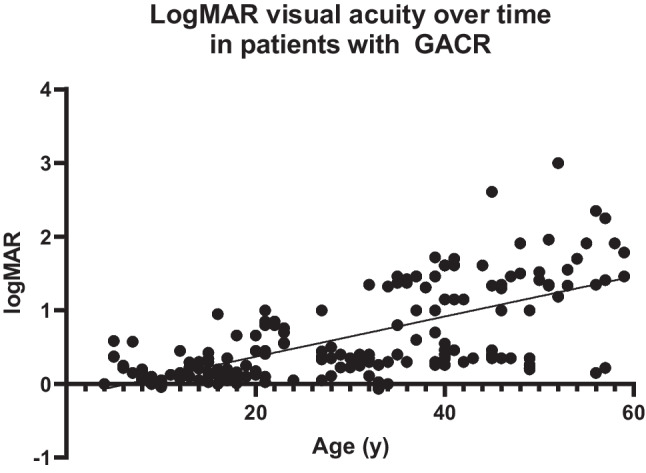
LogMAR values depicted as mean of both eyes in 19 patients with GACR compared to the age at which the measurement was performed. A gradual increase in logMAR can be seen, corresponding to a decrease in visual acuity. There is larger spread in older patients

Analysis of 374 logMAR values retrieved from retrospective chart analysis confirmed the correlation between an older age and worse visual acuity (Fig. 1). Spearman’s ρ was significant at *p* < 0.001 with a correlation coefficient of 0.563.

#### Cataract and cataract surgery

At inclusion, 58% of patients (*n* = 11) were pseudophakic in both eyes. The mean age patients at which underwent cataract surgery was 28.8 years (SD 6.0, range 17–38). No intra- or postoperative complications were reported.

#### Refraction

Mean SE of the refractive error (SER) was -8.96 dioptres (SD 5.11, range -20.87 to -2.25). Pearson correlation showed a significant correlation between age at the time of measurement and SER (*p* < 0.001) with a correlation coefficient of -0.641.

#### Visual field

Humphrey Field Analyser (HFA) 10–2 was used to determine visual field and foveal sensitivity in 12 patients (22 eyes) at inclusion. In five patients (10 eyes), the central ten degrees of the visual field were intact. These patients were all < 30 years (range 11–29). Mean residual visual field in the remaining 12 eyes was 6.08 degrees (SD 2.35, range 1–8).

Mean foveal sensitivity in these 22 eyes was 33.23 dB (SD 4.78, range 23.00–39.00). The mean ‘mean deviation’ (MD) in these eyes was -11.78 (SD 9.31, range -31.83 to -1.11). Pearson’s correlation determined a significant correlation (*p* = 0.003) between foveal sensitivity and corresponding logMAR values of the eyes with a correlation coefficient of -0.606.

**Fig. 2:**
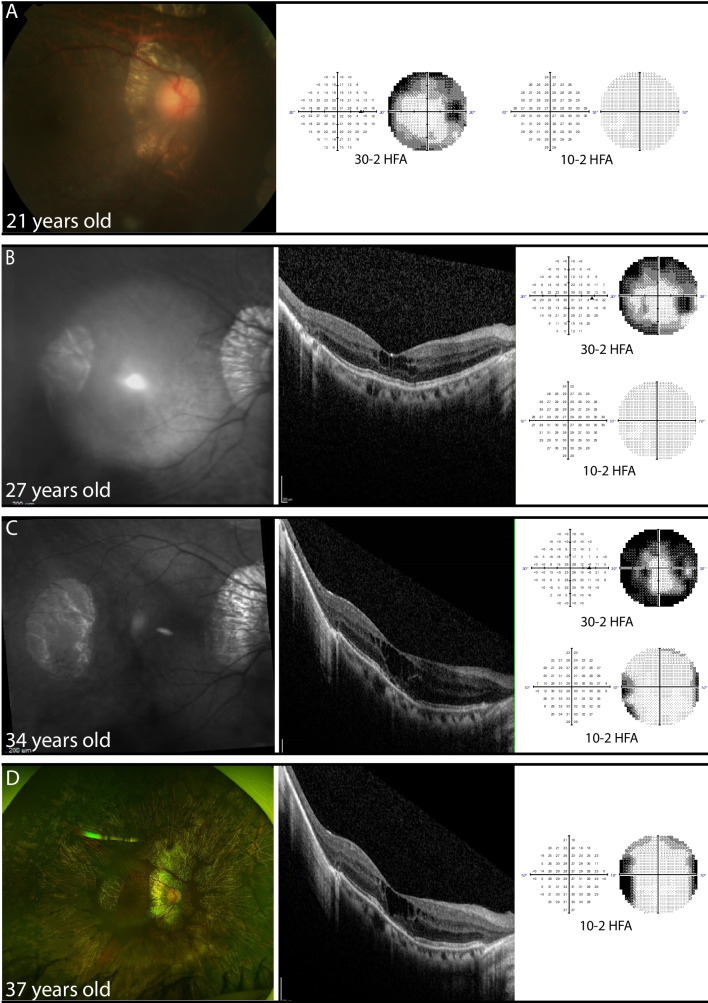
Clinical course of GACR in P1 over the course of 16 years. Images are from OD taken at ages 21 (**A**), 27 (**B**), 34 (**C**) and 37 (**D**). Fundus imaging including retina colour photograph (**A**), IR image (**B**,**C**), and ultra-wide field fundus imaging (**D**). Visual field measurements including 30–2 HFA (**A**, **B**, **C**) and 10–2 HFA (**A**, **B**, **C**, **D**). OCT images (**B**, **C**, **D**). The panels depict a loss of visual field over 16 years, which is especially clear on 30–2 HFA

Retrospective HFA could be retrieved for 5 patients. The follow-up time ranged from 2–16 years, and the median number of available relevant visual fields was 4. Of P1, 16 years of follow-up was present, depicting clear constriction of visual fields over time on HFA (Fig. 2).

### Optical coherence tomography

At inclusion, cystoid maculopathy was present in 68% of patients (*n* = 13, 22/38 eyes), of which 16/22 eyes showed foveal involvement. In 48% of patients (*n* = 9), cystoid maculopathy was present in both eyes. Five patients were receiving treatment for cystoid maculopathy, usually in the form of topical carbonic anhydrase inhibitors.

At inclusion, the foveal EZ was continuous in 14/38 eyes, whereas the perifoveal EZ was continuous in 17/38 eyes. In 9/38 eyes, the foveal EZ was discontinuous, and in 15/38 eyes, it was indiscernible. This is comparable to the perifoveal EZ, where it was discontinuous in 8/38 eyes, and indiscernible in 13/38 eyes. More information on SD-OCT parameters can be found in Table S1 (Supplementary Materials).

For several patients, retrospective SD-OCT imaging was available. In three patients, a progressive loss of EZ became evident between ages 25 and 40 years. Four patients for whom SD-OCT follow-up was available already had advanced disease with an indiscernible foveal EZ on the first available SD-OCT, and these patients where all older than 30 at times of their first available SD-OCT examination. Two patients, P4 and P5, who were both < 18 years at the time of their most recent SD-OCT exam, retained a continuous EZ both in the fovea and perifovea during a follow-up period of 3–5 years.

### Dietary restriction of natural protein

At inclusion, fourteen patients reported adhering to a natural protein-restricted diet during their lifetime. The mean age at which patients started to follow a natural protein-restricted diet was 14.3 years (SD 11.0, range 1–39). The mean duration of dietary therapy was 12.6 years (SD 9.5, range 1–32). Eleven out of fourteen patients were < 18 years old when they started adhering to a natural protein-restricted diet.

The median amount of daily natural protein that treated patients consumed ranged from 0.2–0.8 g/kg/day. Six patients started adhering to a natural protein-restricted diet ≤ 10 years of age. Of these patients, one did not have any fundoscopic lesions during the most recent visit (P13), and three had lesions but full ten degrees of visual field on HFA with BCVA 0.7–1.2 at ages 11–24 (P4, P9, P10).

## Discussion

This cross-sectional study provides an overview of genetic and clinical characteristics in 19 patients with GACR. Although other cohorts have been described [8, 15], this is the first study that provides a standardised ophthalmic assessment of 13 GACR patients. It demonstrates the severe ophthalmic course of GACR, which includes the presence of high myopia, early bilateral cataract, and early loss of EZ integrity. An important limitation of this study is that imaging protocols varied as not all patients could be extensively cross-sectionally screened. In addition, due to severe visual impairment, not all patients could complete all examinations. Finally, several measurements of P3 had to be excluded due to concurrent uveitis.

Several phenotypic aspects stood out within this study. Within this cohort, all patients exhibited myopia, of which 11/19 patients had high myopia with a mean SER of -8.96. This is in strong contrast to the mean SER of -0.10 D which was described by Hendriks et al. in a group of RPE-related dystrophies (*n* = 77), with a prevalence of high myopia in < 10% of the population [16].

In agreement with our results, myopia has been consistently described in many reported GACR cases, although precise refractive errors are often not mentioned [2]. The presence of high myopia is important because it has been shown associated with more rapid disease progression in retinal dystrophies [17–19]. Furthermore, significant association has been established between high myopia and retinal and choroidal changes, such as decreased vessel density and choroidal blood flow [20]. It is not clear why patients with GACR are so much more prone to develop high myopia.

Another highly prevalent feature within this GACR cohort was cataract. The mean age at which patients underwent cataract extraction was 28.8 years, which is in stark contrast to the mean age of cataract surgery in the normal population, which is around 73 years [21].

Cataract is a common anterior segment complication in patients with inherited retinal diseases and tends to be present at a younger age than in patients with age-related cataract [22, 23]. A recent retrospective study reported a mean age of 56.1 years at cataract surgery in a group of retinitis pigmentosa patients (*n* = 226) [23].

There was additional high prevalence of cystoid maculopathy in GACR. At inclusion, cystoid maculopathy was present in 13/19 patients, irrespective of age. It was often treated with carbonic anhydrase inhibitors, although mild cystoid maculopathy usually remained. P5 was the only patient in whom carbonic anhydrase inhibitors resulted in an absence of cystoid maculopathy at follow-up. However, after treatment was stopped, cystoid maculopathy recurred. As systemic carbonic anhydrase inhibitors can cause significant side-effects and have unclear long-term benefit, its use in GACR appears to be of limited importance.

Currently, GACR is mainly treated by restricting dietary natural protein. However, plasma ornithine is rarely normalised despite strict dietary adherence [2]. Although a slowing of disease progression has been described in patients on a natural protein restricted diet, GACR remains progressive, and patients will eventually develop visual impairment [24]. It is difficult to analyse efficacy of protein restriction on ophthalmic outcome measures. In our cohort, the majority of patients was advised to adhere to a natural protein-restricted diet. But there were little comparable outcome measures between patients, and there was a great variation in age and therapeutic compliance. However, four patients who started a protein-restricted diet ≤ 10 years of age still had their central visual fields intact at ages 11–24 years.

To initiate therapies before irreversible damage is done, early diagnosis is essential. Currently, *OAT* is included in many DNA diagnostic gene panels investigating retinal degeneration. This increases the chance of correct diagnosis, especially when a patient is under the attention of an ophthalmologist specialised in retinal degeneration. A genetic diagnosis will not only provide clarity to the patient, but will also provide possible eligibility for future trials, and will increase likelihood of referral to specialised expertise centres for rare disease, thus optimising care.

In conclusion, GACR is a rare disease leading to devastating vision loss. In order to minimise retinal degeneration and vision loss, it is essential to develop new therapeutic interventions. These include gene therapy [11], but also drug repurposing and nutritional therapies. The characterisation of the ophthalmic phenotype as described in this research paper enhances trial readiness. It also provides data on dietary natural protein restriction and helps in establishing reliable and relevant outcome data.

## Supplementary Information

Below is the link to the electronic supplementary material.

Supplementary file1 (DOCX 800 KB)
