# Supplementary material for: A cohort study of 19 patients with gyrate atrophy of the choroid and retina (GACR)
Source: Graefes Arch Clin Exp Ophthalmol. 2024 Jun 7;262(11):3589–96. doi: 10.1007/s00417-024-06540-8 (PMC11584430; doi:10.1007/s00417-024-06540-8)
Supplement: Supplementary file 1 — Supplementary file1 (DOCX 800 KB) [file 417_2024_6540_MOESM1_ESM.docx]

**Supplemental File**

**Table S1: CME treatments and optical coherence tomography at most recent visit; years of follow-up prior to recent visit and progression within that time.**

| **ID** | **Eye** | **CME** | **Fovea-involving** | **Epiretinal membrane** | **Foveal EZ integrity** | **Perifoveal EZ integrity** | **Current CME treatment** | **Previous CME treatments** | **Years of OCT follow-up** | **Progression** |
| --- | --- | --- | --- | --- | --- | --- | --- | --- | --- | --- |
| P1 | OD | Yes | Yes | No | Indiscernible | Continuous | Brinzolamide  1 drop 2x/day | None | 10 | Yes, loss of foveal EZ integrity |
|  | OS | Yes | Yes | No | Indiscernible | Continuous |  |  |  |  |
| P2 | OD | No | NA | No | Indiscernible | Indiscernible | None | Unknown | 6 | No |
|  | OS | No | NA | Yes | Indiscernible | Indiscernible |  |  |  |  |
| P3 | OD | Yes | Yes | Yes | Indiscernible | Indiscernible | None | Acetazolamide 125 mg 2x/day  Octreotide 30 mg 1x/4 weeks  Dorzolamide 1 drop 3x/day | NA* | NA |
|  | OS | Yes | Yes | Yes | Indiscernible | Indiscernible |  |  |  |  |
| P4 | OD | Yes | No | No | Continuous | Continuous | Dorzolamide  1 drop 3x/day | Brinzolamide 1 drop 2x/day | 5 | Yes, development of epiretinal membrane OS |
|  | OS | Yes | No | Yes | Continuous | Continuous |  |  |  |  |
| P5 | OD | No | NA | No | Continuous | Continuous | None | Brinzolamide  1 drop 3x/day | 3 | Yes, recurrence of CME ODS |
|  | OS | Yes | Yes | no | Discontinuous | Continuous |  |  |  |  |
| P6 | OD | No | NA | No | Continuous | Continuous | None | None | NA | NA |
|  | OS | No | NA | No | Continuous | Continuous |  |  |  |  |
| P7 | OD | No | No | No | Indiscernible | Indiscernible | None | Brinzolamide 1 drop 2x/day  Timolol/latanoprost 1 drop 1x/day | 5 | No |
|  | OS | Yes | Yes | No | Indiscernible | Indiscernible |  |  |  |  |
| P8 | OD | Yes | Yes | No | Indiscernible | Indiscernible | Acetazolamide  125 mg 2x/day | Acetazolamide 250 mg 2x/day  Acetazolamide 250 mg 1x/day | NA | NA |
|  | OS | Yes | Yes | No | Indiscernible | Indiscernible |  |  |  |  |
| P9 | OD | Yes | No | No | Continuous | Continuous | None | Acetazolamide | NA | NA |
|  | OS | Yes | Yes | No | Continuous | Continuous |  |  |  |  |
| P10 | OD | Yes | No | No | Continuous | Continuous | None | None | NA | NA |
|  | OS | Yes | Yes | No | Continuous | Continuous |  |  |  |  |
| P11 | OD | Yes | No | Yes | Discontinuous | Discontinuous | Acetazolamide  125 mg 2x/day | Acetazolamide 250 mg 2x/day  Octreotide 20 mg 1x/4 weeks  Vitrectomy ODS | 7 | Yes, loss of (peri)foveal EZ integrity, remission and recurrence of CME |
|  | OS | Yes | Yes | Yes | Discontinuous | Discontinuous |  |  |  |  |
| P12 | OD | No | NA | No | Discontinuous | Discontinuous | None | None | NA | NA |
|  | OS | No | NA | No | Discontinuous | Discontinuous |  |  |  |  |
| P13 | OD | No | NA | No | Continuous | Continuous | None | None | NA | NA |
|  | OS | No | NA | No | Continuous | Continuous |  |  |  |  |
| P14 | OD | Yes | Yes | No | Discontinuous | Discontinuous | Dorzolamide/timolol  1 drop 2x/day | Acetazolamide 250 mg 2x/day  Dorzolamide 1 drop 2x/day  Brimonidine 1 drop 2x/day | 7 | Yes, loss of (peri)foveal EZ integrity |
|  | OS | Yes | Yes | No | Discontinuous | Discontinuous |  |  |  |  |
| P15 | OD | Yes | Yes | No | Discontinuous | Discontinuous | None | None | NA | NA |
|  | OS | No | NA | No | Continuous | Continuous |  |  |  |  |
| P16 | OD | Yes | No | No | Continuous | Continuous | None | None | NA | NA |
|  | OS | No | NA | No | Continuous | Continuous |  |  |  |  |
| P17 | OD | No | NA | No | Discontinuous | Discontinuous | None | None | 12 | Yes, loss of (peri)foveal EZ integrity OD |
|  | OS | No | NA | No | Indiscernible | Indiscernible |  |  |  |  |
| P18 | OD | Yes | Yes | No | Indiscernible | Indiscernible | None | None | 2 | No |
|  | OS | Yes | Yes | No | Discontinuous | Discontinuous |  |  |  |  |
| P19 | OD | No | NA | No | Indiscernible | Indiscernible | None | None | 6 | No |
|  | OS | No | NA | No | Indiscernible | Indiscernible |  |  |  |  |

*OCT imaging was excluded from analyses due to the presence of posterior uveitis ODS.

**Table S2: Pathogenic variants identified including ACMG classifications and novelty of variants**

| **Variant** | **ACMG classification** | **Reported previously^*^** |
| --- | --- | --- |
| c.1A>G; p.(1M) | Class 4 (likely pathogenic) | Yes |
| c.1058G>A; p.(Gly353Asp) | Class 5 (pathogenic) | Yes |
| c.1276C>T; p.(Arg426Ter) | Class 5 (pathogenic) | Yes |
| c.460C>T; p.(Arg154Cys) | Class 3 (uncertain significance) | Yes |
| c.539G>C; p.(Arg180Thr) | Class 4/5 (likely pathogenic/pathogenic) | Yes |
| c.1034T>C; p.(Leu345Pro) | Class 4 (likely pathogenic) | No |
| c.1112dup; p.(Arg372Lysfs*12) | Class 4 (likely pathogenic) | Yes |
| c.1172delT; p.(Trp391Glyfs*29) | Class 4/5 (likely pathogenic/pathogenic) | Yes |
| c.1235T>C; p.(Ile412Thr) | Class 4 (likely pathogenic) | No |
| c.1173G>A; p.(Trp391Ter) | Class 4/5 (likely pathogenic/pathogenic) | Yes |
| c.1192C>T; p.(Arg398*) | Class 5 (pathogenic) | Yes |

*Reported in the GnomAD database.

**Figure S1: Optical coherence tomography (OCT) of several different patients with GACR depicting the ellipsoid zone (EZ) as continuous, discontinuous, or indiscernible.**


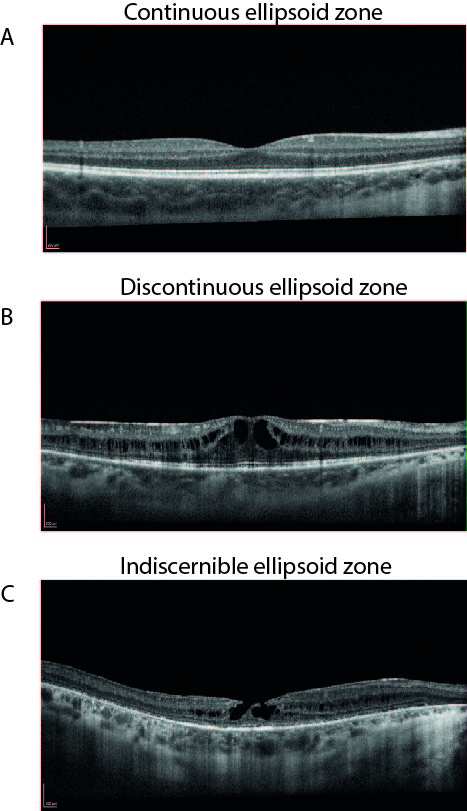


*A) OCT of an 11-year-old patient with GACR with a continuous EZ. B) OCT of a 28-year-old patient with GACR with a discontinuous (foveal) EZ. C) OCT of a 49-year-old patient with GACR with an indiscernible EZ.*
